# Solute carrier transporter disease and developmental and epileptic encephalopathy

**DOI:** 10.3389/fneur.2022.1013903

**Published:** 2022-11-07

**Authors:** Yajing Gan, Zihan Wei, Chao Liu, Guoyan Li, Yan Feng, Yanchun Deng

**Affiliations:** ^1^Department of Neurology, Epilepsy Center of Xijing Hospital, Fourth Military Medical University, Xi'an, China; ^2^Xijing Institute of Epilepsy and Encephalopathy, Xi'an, China

**Keywords:** SLC, DEE, epilepsy, treatment, pathogenesis

## Abstract

The International League Against Epilepsy officially revised its classification in 2017, which amended “epileptic encephalopathy” to “developmental and epileptic encephalopathy”. With the development of genetic testing technology, an increasing number of genes that cause developmental and epileptic encephalopathies are being identified. Among these, solute transporter dysfunction is part of the etiology of developmental and epileptic encephalopathies. Solute carrier transporters play an essential physiological function in the human body, and their dysfunction is associated with various human diseases. Therefore, in-depth studies of developmental and epileptic encephalopathies caused by solute carrier transporter dysfunction can help develop new therapeutic modalities to facilitate the treatment of refractory epilepsy and improve patient prognosis. In this article, the concept of transporter protein disorders is first proposed, and nine developmental and epileptic encephalopathies caused by solute carrier transporter dysfunction are described in detail in terms of pathogenesis, clinical manifestations, ancillary tests, and precise treatment to provide ideas for the precise treatment of epilepsy.

## Introduction

The concept of epileptic encephalopathy was widely accepted and used in the early years, but later scholars have found that many genetic disorders can cause developmental disorders in addition to the direct effects of epileptic activity on development (1). In 2017, the International League Against Epilepsy (ILAE) introduced the concept of developmental and epileptic encephalopathy (DEE) (2). Most DEEs have a genetic cause. With the advances in genetic testing and neuroimaging in recent years, an increasing number of variants of new genes have been discovered, advancing the understanding of DEE etiology. Transporter dysfunction accounts for one etiology of DEE, with solute carrier (SLC) transporters predominating. SLC transporters not only play an essential role in maintaining human physiological functions but also are important targets for drug action in the human body, and in-depth analysis of SLC transporters can significantly contribute to the development of treatments for refractory epilepsy. To date, nine SLC genes related to DEE have been identified. The study of the pathogenesis of these nine types of DEEs will improve targeted treatment and the prognosis of patients.

## Transporter proteins and transporter protein diseases

Transporter proteins are widely expressed on human cellular and organelle membranes. It is believed that 10% of human genes are associated with transport functions, which control the passage of substances across membranes and are essential for cell growth and reproduction (3). In total, four superfamilies of transporter proteins have been identified: the ATP-binding cassette (ABC) superfamily, ATPase superfamily, ion channel superfamily, and SLC superfamily (4). The ATP-binding cassette and SLC superfamilies are the main components of transporter proteins. SLCs are present in almost every organelle and cell membrane and play a significant role in maintaining environmental homeostasis in the human body. Broadly speaking, we refer to all diseases caused by variants of genes encoding transporter proteins as transporter protein diseases, and narrowly speaking, for those mainly caused by variants of the SLC transporter genes, we call them SLC transporter diseases.

## Solute carrier transporter disease

There are 458 different transmembrane SLCs in humans, which are categorized into 65 families (5). SLC transporters, one of the major mammalian cell membrane proteins, are involved in a wide range of transport functions between various organelles. Since they rely on electrochemical gradients or ion gradients generated by proton pumps to transport substrates, SLC transporters are considered secondary active transporter proteins (6). In the brain, SLC transporters are involved in the transport of various solutes across membranes, such as across the blood–brain barrier, cell membranes, and mitochondrial membranes. This transport plays an essential role in maintaining normal brain function, and SLC transporter dysfunction can contribute to various neurological diseases, such as Huntington's disease, Parkinson's disease, Alzheimer's disease, and epilepsy (7, 8). Furthermore, an increasing number of monogenic diseases have been found to be associated with SLC, suggesting that transporter proteins have very high potential for exploitation. In this article, we only describe the association of SLC with DEE because an in-depth study of SLC may reveal some precise therapeutic targets that could promote the development of treatments for drug-refractory epilepsy. Based on the literature, we found that variants of *SLC1A2, SLC2A1, SLC6A1, SLC12A5, SLC13A5, SLC25A22, SLC35A2*, and *SLC38A3* are associated with a series of DEEs, and the pathogenesis and precise therapeutic measures for each of these nine DEEs are described in the following text.

## Pathogenesis, clinical manifestations, and treatment of solute carrier transporter diseases

### *SLC1A2* and developmental and epileptic encephalopathy type 41

#### Pathogenesis

**Figure 1:**
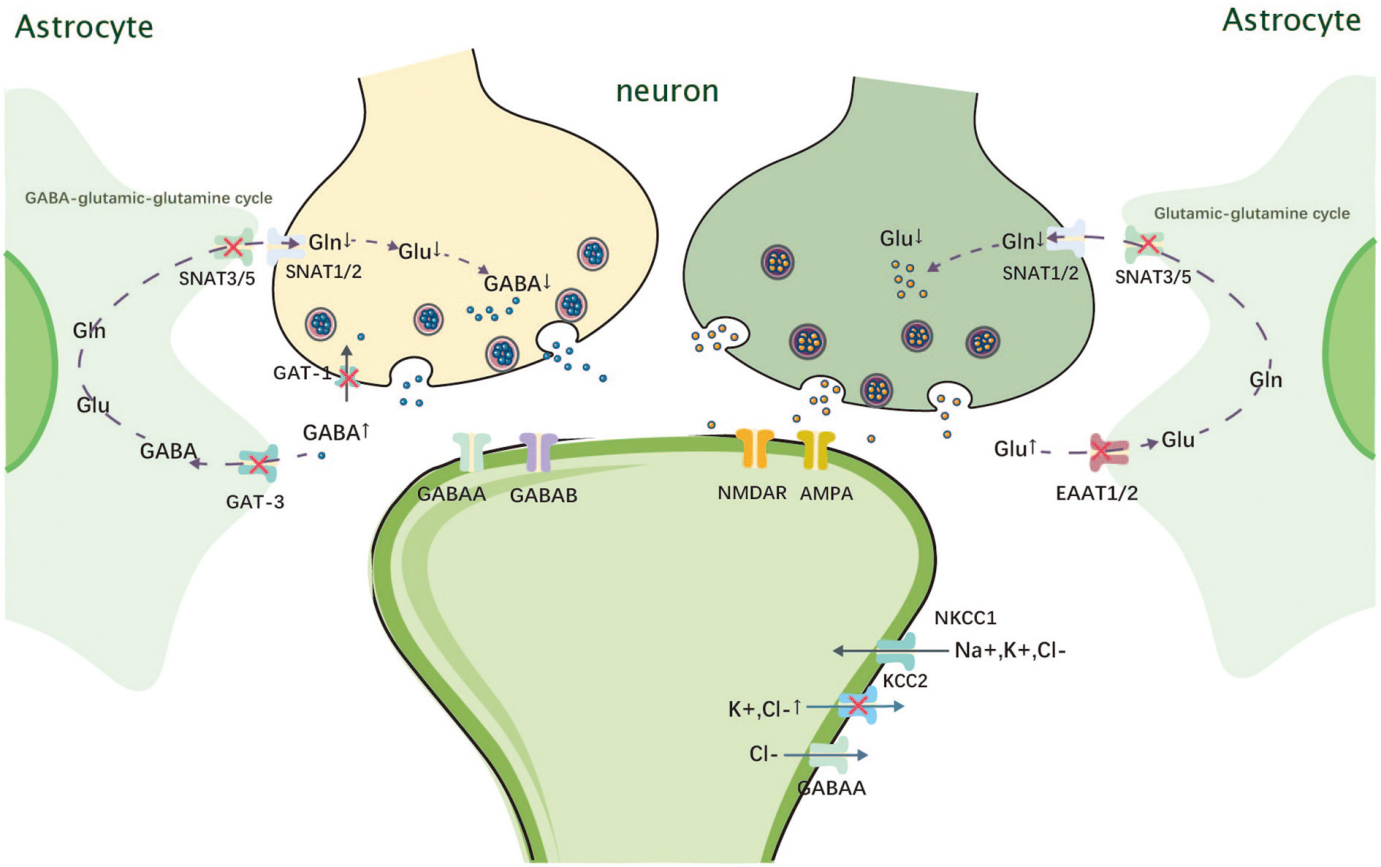
Mechanism of action of *SLC1A2, SLC6A1, SLC12A5*, and *SLC38A3*. Gln, glutamine; Glu, glutamate; GABA, gamma-amino butyric acid; SNAT, sodium-coupled neutral amino acid transporter; NMDAR, N-methyl-D-aspartate receptor; AMPA, alpha-amino-3-hydroxy-5-methyl-4 -isoxazolepropionic acid receptor; GAT-1, Na+-Cl–coupled γ-aminobutyric acid transporter 1; NKCC1, Na+-K+-2Cl- cotransporter 1; KCC2, K+-Cl-cotransporter 2; EAAT, excitatory amino acid transporter.

The SLC1 family mediates the cellular uptake of glutamate, thus reducing the intersynaptic glutamate concentration and helping to terminate excitatory neurotransmission (9). *SLC1A2* is a member of the SLC1 family located on chromosome 11p13 and encodes sodium-dependent glutamate transporter 2, which is also known as excitatory amino acid transporter 2 (EAAT2) (10). Pathogenic variants of *SLC1A2* can lead to developmental and epileptic encephalopathy type 41 (DEE41, OMIM 600300) (11). EAAT2 is expressed on the surface of astrocytes and is responsible for transporting more than 80–90% of glutamate by the codirectional transfer of three Na^+^ and one H^+^ ions and receiving one K^+^ ion in exchange (9). EAAT2 reduces the extracellular concentration of glutamate, thus preventing excessive stimulation of cell membrane receptors by glutamate, which can lead to neuronal damage or death (Figure 1) (12, 13).

#### Clinical presentation

Variants of *SLC1A2* are autosomal dominant, most of which are heterozygous *de novo* variants (14). Wagner et al. found that autosomal recessive inherited double-allele variants of *SLC1A2* can also cause epilepsy (15). All patients with autosomal dominant inheritance of *SLC1A2* developed seizures within the first 6 months of life (16). They typically begin as tonic or myoclonic seizures and then gradually evolve into multiple seizure types, such as atonic seizures, tonic–clonic seizures, and generalized tonic seizures, with global developmental delay, axial hypotony, optic nerve atrophy, and cortical visual dysplasia (16). The homozygous splicing variant reported by Wagner et al. had less severe symptoms, with the first seizure at the age of 2 years, starting with vomiting and nervous eye deviation, followed by generalized tonic–clonic and atonic seizures (15). This patient also had autism and attention-deficit/hyperactivity disorder (ADHD), and a single medication was sufficient to bring the developmental delay under control (15). The EEG of most patients showed multifocal or diffuse epileptiform discharges with abnormal background activity (16). EEG of patients with autosomal recessive inheritance showed diffuse hemispheric dysfunction (15).

#### Treatment options

DEE41 is a treatment-refractory epilepsy, while DEE41 in one patient was well controlled with valproic acid monotherapy (17). Translational activators of EAAT2 may provide a new therapeutic approach to protect neurons from excitatory neurotransmitter damage (12, 18). Dyomina et al. showed that a recombinant form of the interleukin 1 receptor antagonist (anakinra) reduced neuronal loss in the hippocampal region and enhanced EAAT2 expression levels, thereby suppressing recurrent seizures in the chronic phase (19). This may also prevent some behavioral disorders, such as motor hyperactivity and disturbances in social interactions (19). A pyrazine derivative named LDN/OSU-0212320, identified by Kong et al., can reduce excitotoxic effects and increase glutamate uptake by glial cells by upregulating EAAT2 translation (20). LDN/OSU-0212320 was found to significantly reduce neuronal death and seizure frequency in mice (20). Parawixin10 is an EAAT1 and EAAT2 allosteric modulator, which has neuroprotective effects and reduces the severity and duration of seizures (18). However, tamoxifen and growth factor α-selective agonists enhance EAAT1 and EAAT2 expression through the activation of the NF-κB pathway (21, 22). In addition, ceftriaxone, a β-lactam antibiotic, is one of the well-studied *SLC1A2* modulators, and it is an EAAT2 protein enhancer that reduces neuronal death and seizure frequency (23, 24). An animal study also showed that low doses of ceftriaxone with valproate increased GABAergic activity and decreased glutamatergic activity, favoring seizure suppression and improved cognitive function (25). However, Stergachis et al. did not find significant improvements in *SLC1A2-*associated epilepsy in 20-month-old patients treated with ceftriaxone (16).

### *SLC2A1* and glucose transporter 1 deficiency syndromes 1 and 2

#### Pathogenesis

The SLC2 family plays an important role in the control of systemic glucose homeostasis (26). The *SLC2A1* gene belongs to the first identified member of the SLC2 family of glucose transporters and encodes glucose transporter 1 (GLUT1). GLUT1 is the main transporter protein that mediates glucose crossing the blood–brain barrier. GLUT1 deficiency not only decreases glucose transport in the brain but also prevents cerebral angiogenesis, resulting in a smaller than normal brain microvasculature (27). Both brain glucose deficiency and a smaller microvasculature deprive the brain of the energy resources it needs to function properly.

#### Clinical presentation

GLUT1 dysfunction can lead to glucose transporter 1 deficiency syndrome (GLUT1DS), including GLUT1DS1 (OMIM 606777) and GLUT1DS2 (OMIM 612126). Approximately 90% of GLUT1DS is caused by *de novo* heterozygous variants, whereas GLUT1DS homozygote variants can be morphologically abnormal during the embryonic stage and are incompatible with life (28).

GLUT1DS1 is mainly autosomal dominant and, to a lesser extent, autosomal recessive. Approximately 80% of patients develop seizures in infancy, which can present as generalized tonic–clonic, myoclonic, atonic, or atypical absence seizures (29, 30). Patients mostly present with microcephaly, hypotony, dystonia, ataxia, hyperreflexia, developmental delay, mental retardation, and motor disorders such as paroxysmal hemiparesis, paroxysmal limb stiffness and purple limb, paroxysmal oculomotor abnormalities, and chorea (29, 31).

GLUT1DS2 is autosomal dominant, and its main phenotype is paroxysmal exercise-induced dyskinesia (PED), which commonly develops concurrently with prolonged exercise as the main trigger but can also be triggered by anxiety and emotional stress in a few patients (32). The seizures may be preceded by autonomic symptoms such as hyperventilation and sweating (33). Some patients also have progressive spastic paraplegia, paroxysmal chorea, paroxysmal limb weakness, ataxia, developmental delay, dysarthria, and epilepsy (31, 34, 35). Their epilepsy mainly manifests as absence and, to a lesser extent, as atypical absence seizures, myoclonic seizures, and atonic seizures (33, 36).

It is worth noting that GLUT1DS has significantly lower epileptiform abnormalities on postprandial EEG than on preprandial EEG (37, 38). MRIs are mostly normal or mildly abnormal, including mild brain atrophy, myelin dysplasia, ventricular enlargement, and corpus callosum dysplasia (39). 18F-fluorodeoxyglucose positron emission tomography (18F-FDG-PET) showed reduced glucose metabolism in the bilateral thalamus, cerebellum, and neocortex, which may be associated with epilepsy (40). Cerebrospinal fluid lactate glucose measurement can be a rapid and effective method to diagnose GLUT1DS because almost all patients with GLUT1DS have reduced glucose levels in the cerebrospinal fluid and normal or reduced lactate levels (41).

#### Treatment options

The ketogenic diet is a very effective tool that increases blood ketone levels, and ketones cross the blood–brain barrier *via* monocarboxylate transporter 1 and supply the brain with energy (42–44). Thus, a ketogenic diet can improve seizure symptoms, motor deficits, and cognition of most patients (45–47). Triheptanoin is metabolized to acetyl coenzyme A and propionyl coenzyme A, which can stimulate the tricarboxylic acid cycle and effectively improve brain energy deficiency in patients with GLUT1DS (48). Joshi et al. reported a case of a boy with partial seizure control through the use of a medium-chain triglyceride oil-based ketogenic diet (35). The addition of carnitine and alpha-lipoic acid resulted in complete seizure control and significant improvements in motor and cognitive function (35). Phenobarbital and valproic acid inhibit GLUT1-DS transport and should be avoided (49). A study by Tang et al. showed that using adeno-associated virus serotype 9 (AAV9) as a vector for *SLC2A1* in model mice increased GLUT1 expression in the mouse brain, increased the cerebrospinal fluid glucose concentration, increased glucose uptake in the brain, and restored the brain microvessel size (50). Directly combining GLUT1 with transduction domains or cell-penetrating peptides for delivery into cells is a possible new therapy (51).

### *SLC6A1* and myoclonic–atonic epilepsy

#### Pathogenesis

*SLC6A1* belongs to the γ-aminobutyric acid (GABA) transporter subfamily, is the most abundantly expressed GABA transporter protein in the central nervous system (CNS), and is one of the most intensively studied members of the SLC6 family (52). It is located on chromosome 3p25.3, encoding Na^+^-Cl^−^-coupled γ-aminobutyric acid transporter 1 (GAT-1), which is mainly responsible for the reuptake of GABA from the synapse (Figure 1). GAT-1 dysfunction leads to GABA accumulation between synapses and overstimulation of GABAA and GABAB receptors on the postsynaptic membrane (42). GABAA overstimulation can cause continuous hyperpolarization and outbreak discharge of cell membranes, which cause seizures (43).

#### Clinical presentation

*SLC6A1* pathogenic variants cause myoclonic–atonic epilepsy (OMIM 616421), but there is no interaction between seizures and cognitive impairment (44). The main clinical features also include cognitive developmental impairment, mild to moderate intellectual disability, autism spectrum disorders, and language disorders. However, a small number of patients carrying *SLC6A1* variants do not have seizures and show only mild intellectual disability, suggesting that the expression of the gene is related to the environment and is subject to epigenetic modifications (44, 53). The EEG of patients with the *SLC6A1* variant may have some similar features, such as the appearance of irregular spikes, multiple spikes, slow waves over a wide range of 2.5–3.5 Hz, and slowed background activity in the EEG (52). MRI does not show specific results and may show enlarged frontal lobe gaps and cerebellar vermis hypoplasia (44).

#### Treatment options

A ketogenic diet makes astrocytes metabolically active and increases the amount of glutamine in the brain, allowing for a more efficient conversion of glutamine to GABA, which exerts an antiepileptic effect (54, 55). Seizures can be partially or even completely controlled with valproate monotherapy or in combination with other antiepileptic drugs in patients with epilepsy (44, 56). This may be related to the fact that valproate increases the concentration of the neurotransmitter GABA in the brain and reduces the excitability of neurons (57). Tiagabine is a selective inhibitor of GAT-1 in the brain, and vigabatrin is a GABA amino transaminase, both of which act by increasing the concentration of GABA in the brain (20, 58). Since *SLC6A1* can be packaged in an AAV9 vector, AAV-mediated gene therapy shows good therapeutic promise (59). Antisense oligonucleotide treatment also specifically increases GAT-1 protein (60). Nwosu et al. found that patients with *SLC6A1* pathogenic variants are heterozygous and that all carry wild-type GAT-1 (61). 4-Phenylbutyrate alone can increase GAT-1 expression and promote positive transport of wild-type GAT-1, thereby increasing GABA reuptake by astrocytes with *SLC6A1*-mutant neurons (61). Thus, targeting GAT-1 could open new avenues to treat refractory epilepsy.

### *SLC12A5* and developmental and epileptic encephalopathy type 34

#### Pathogenesis

The SLC12 family encodes a neutral cation–chloride cotransporter protein involved in transepithelial ion transport, which is associated with the regulation of cell volume and intracellular chloride ion concentration (62). The *SLC12A5* gene located on chromosome 20q13.12 encodes mammalian K^+^-Cl^−^ cotransporter 2 (KCC2), which is expressed only in neurons (63). However, the Na^+^-K^+^-2Cl^−^ cotransporter (NKCC1) encoded by the *SLC12A2* gene is widely expressed in the brain (64). GABA is the most important inhibitory neurotransmitter in the CNS, whose function depends on KCC2 activity. KCC2 maintains a low intracellular chloride ion concentration, which helps improve GABA and glycine function, whereas NKCC1 has the exact opposite function (Figure 1) (65, 66). The effect of GABA depends on the intracellular chloride concentration: when the intracellular chloride concentration is too high, GABA switches from an inhibitory to an excitatory transmitter (67). According to the current literature, NKCC1 is gradually downregulated as development progresses, while KCC2 expression increases (64). Therefore, during the early stages of development, GABAergic neurotransmission can lead to chloride efflux and membrane depolarization due to high NKCC1 expression levels and high intracellular chloride ion concentrations (67). As the brain develops, the expression of KCC2 gradually increases, causing a shift from GABAergic excitation to inhibition (68). When KCC2 is dysfunctional, the excitatory–inhibitory balance of GABA is disrupted, which may lead to the development of epilepsy (69, 70).

#### Clinical presentation

Variants of *SLC12A5* can lead to developmental epileptic encephalopathy type 34 (DEE34) (OMIM 616645) and idiopathic generalized epilepsy (OMIM 616685). Few cases of DEE34 have been reported, and the phenotype can be consistent with epilepsy of infancy with migrating focal seizures (EIMFS). The clinical features are mainly seizures within the first 6 months of life, developmental delay or regression, microcephaly, and low eye pressure (71, 72). It mainly starts with focal epilepsy and gradually changes to multifocal seizures that are unresponsive to antiseizure medications (71, 72). Patients with EIMFS have EEGs that are typical of migrating seizures, with regular background waves at the end of the episode, followed by slow-wave activity (71, 72). Some patients' MRI may present with myelin retardation, brain atrophy, and corpus callosum thinning (71).

#### Treatment options

DEE34 in general is treatment-refractory epilepsy, but treatment with a ketogenic diet and potassium bromide has been reported to reduce the seizure frequency in some patients (66, 72). Bumetanide is an NKCC1 inhibitor, and its specific mechanism of action remains unclear. Bumetanide is effective in refractory temporal lobe epilepsy, but some patients have experienced adverse effects, such as loss of appetite, nausea, vomiting, and increased seizure frequency (73). Studies by Kahle et al. have also shown that bumetanide significantly reduces the duration and frequency of seizures in neonates (74). However, in an open-label clinical trial, bumetanide as an add-on treatment to phenobarbital did not improve seizures in neonates with hypoxic ischemic encephalopathy and even increased the risk of hearing damage (75). Therefore, the effectiveness and safety of bumetanide need further study (76, 77). Stimulation of IGF-1 receptors accelerates activation of the KCC2 protein, so IGF-1 can increase KCC2 expression and decrease the NKCC1/KCC2 ratio (78). In neurons, the WNK-SPAK/OSR1 pathway regulates NKCC1 and KCC2, and inhibition of this pathway promotes intracellular chloride ion elimination and GABAergic inhibition, which exerts an antiepileptic effect (79, 80).

### *SLC13A5* and developmental and epileptic encephalopathy type 25

#### Pathogenesis

In mammals, the SLC13 superfamily transports metabolic intermediates of the citric acid cycle in a 3Na+/1 substrate stoichiometry ratio (81, 82). *SLC13A5* is located on chromosome 17p13.1 and encodes Na+/citric acid transporter (NACT) protein. NACT dysfunction can cause developmental epileptic encephalopathy type 25 (DEE25, OMIM 615905) with dental enamel hypoplasia (83). NACT is primarily expressed on the surface of neurons, where Na+ transport provides electrochemical dynamics, which can transport circulating citric acid into neurons, promote the utilization of circulating citric acid, and provide energy to neuronal cells. The exact mechanism by which variants of *SLC13A5* cause epilepsy and neurodevelopmental disorders is still unclear, but researchers have proposed three hypotheses to explain this pathogenesis: (1) Cytoplasmic citric acid deficiency hypothesis: NACT gene variants cause a decrease in the cytoplasmic citric acid content and an insufficient supply of neuronal energy, resulting in epilepsy and delayed brain development (84); (2) interneuron energy hypothesis: in the brain, the metabolic energy consumption of inhibitory neurons is higher than that of excitatory neurons, and *SLC13A5* pathogenic variants make neurons less able to restore ionic gradients, leading to an excitatory–inhibitory imbalance (84); and (3) zinc chelation hypothesis: variants of the *SLC13A5* gene lead to a relative increase in extracellular citric acid concentration, causing partial zinc and NCTA chelation, which enhances NMDA receptor-mediated synaptic transmission and leads to an imbalance in excitatory inhibition (85).

#### Clinical presentation

Variants of *SLC13A5* are autosomal recessive and characterized clinically by seizures in the first weeks of life; most patients present with focal myoclonic seizures and a few with secondary generalized seizures in the neonatal period (86, 87). Patients may also present with dental dysplasia, ataxia, axial hypotony, motor deficits, and developmental delays (86, 87). Dental hypoplasia is a characteristic presentation and can be used as a screening indicator for key populations. Speech impairment is particularly evident in this group of patients and can manifest as altered speech intonation (86, 87). The prognosis of DEE25 varies widely, with most patients having significantly less frequent seizures as they grow up, but some children with DEE25 die during the neonatal period due to persistent epilepsy and respiratory insufficiency (87, 88). Yang et al. studied the EEG characteristics of 23 patients with *SLC13A5* variants and found that the patients in the neonatal period showed frequent epileptiform abnormalities with multifocal spikes and sharps, while the EEG background remained essentially normal after the neonatal period (88). MRI showed characteristic punctate white matter damage and periventricular white matter softening (89). By 6 months of age, the punctate white matter high signal disappeared, followed by glial scarring (89).

#### Treatment options

The efficacy of a ketogenic diet in these patients is uncertain. The three patients reported by Hardies et al. responded well to a ketogenic diet, but some patients who used a ketogenic diet experienced a deterioration of their condition (87, 90). High concentrations of lithium (Li) can enhance the transport function of NACT, and Li can occupy the four sodium-binding sites of NACT, producing a stimulatory effect, but whether it has an antiepileptic effect remains to be verified (84, 91). Acetazolamide controls seizures in some patients and may increase plasma acidity and decrease intracranial pressure by reducing urinary citrate excretion, but the specific antiepileptic effects of acetazolamide are still unclear (90, 92). Antiseizure medications that act through the GABA system, such as valproic acid and phenobarbital, are the most commonly used drugs to treat this disorder, and they significantly decrease the seizure frequency (90). A patient reported by Pellegrino et al. had a significant reduction in seizure frequency and significant improvements in motor skills and speech comprehension after treatment with a combination of valproic acid and acetazolamide (93).

### *SLC25A12* and developmental and epileptic encephalopathy type 39

#### Pathogenesis

**Figure 2:**
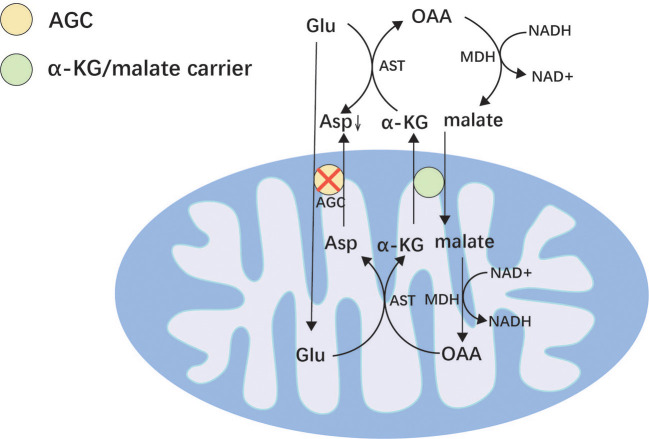
Malate/aspartate shuttle. Mitochondria are impermeable to NADH and transport NADH into the mitochondria *via* the malate/aspartate form to supply the respiratory chain to generate ATP. AGC can reverse the transport of glutamate and aspartate and plays an important role in the malate/aspartate shuttle. Asp, aspartic acid; OAA, oxaloacetic acid; AST, aspartate aminotransferase; MDH, malate dehydrogenase; α-KG, α-ketoglutaric acid; AGC, mitochondrial aspartate/glutamate transporter; Glu, glutamic.

*SLC25A12* is located on chromosome 2q31.1 and encodes a mitochondrial aspartate/glutamate transporter (AGC) (94). *SLC25A12* is expressed mainly in neurons and skeletal muscles and is a major component of the malate/aspartate shuttle (Figure 2) (94, 95). It allows aspartate to be exchanged for glutamate and protons in the mitochondria and transfers their cytosolic reducing equivalents into mitochondria (95). When AGC is dysfunctional, aspartate cannot be transported into the cytoplasm, resulting in reduced production of N-acetyl glutamate in the cytoplasm, which leads to poor myelin production (95, 96). However, the exact mechanism by which variants of *SLC25A12* cause epilepsy remains unclear. It is speculated that it may be related to intracellular glutamate accumulation and cellular damage.

#### Clinical presentation

*SLC25A12* variants are associated with developmental epileptic encephalopathy type 39 (DEE39, OMIM 612949), which is autosomal recessive. In previous studies, four patients have been reported, and their main clinical features are early-onset epilepsy, psychomotor retardation, hypotonia, short stature, and microcephaly (96–98). Brain MRI shows a characteristic myelin developmental disorder and brain atrophy (97). Proton magnetic resonance wave analysis may show a reduced N-acetyl aspartate peak, which indicates neuronal damage (96).

#### Treatment options

A ketogenic diet significantly controls seizures in AGC1 deficiency, suggesting that a ketogenic diet may be linked to the pathogenesis of AGC1 deficiency. (1) A ketogenic diet provides acetyl coenzyme A directly to the mitochondrial tricarboxylic acid cycle, ameliorating neuronal energy deficiency (99). (2) A ketogenic diet reduces NADH production from cellular glycolysis, and malate dehydrogenase is an NADH-dependent dehydrogenase, so oxaloacetate is converted into aspartate, rather than malate, promoting neuronal use of aspartate and N-acetyl glutamate for myelin production (100). (3) β-Hydroxybutyrate has a protective effect on neurons and restores mitochondrial respiration, increasing cytoplasmic aspartate and N-acetyl glutamate, which can promote myelin formation (101). In previous studies, two patients with *SLC25A12* pathogenic variants had a dramatic decrease in seizure frequency and some improvement in psychomotor development after adopting a ketogenic diet (99, 100).

### *SLC25A22* and developmental and epileptic encephalopathy type 3

#### Pathogenesis

**Figure 3:**
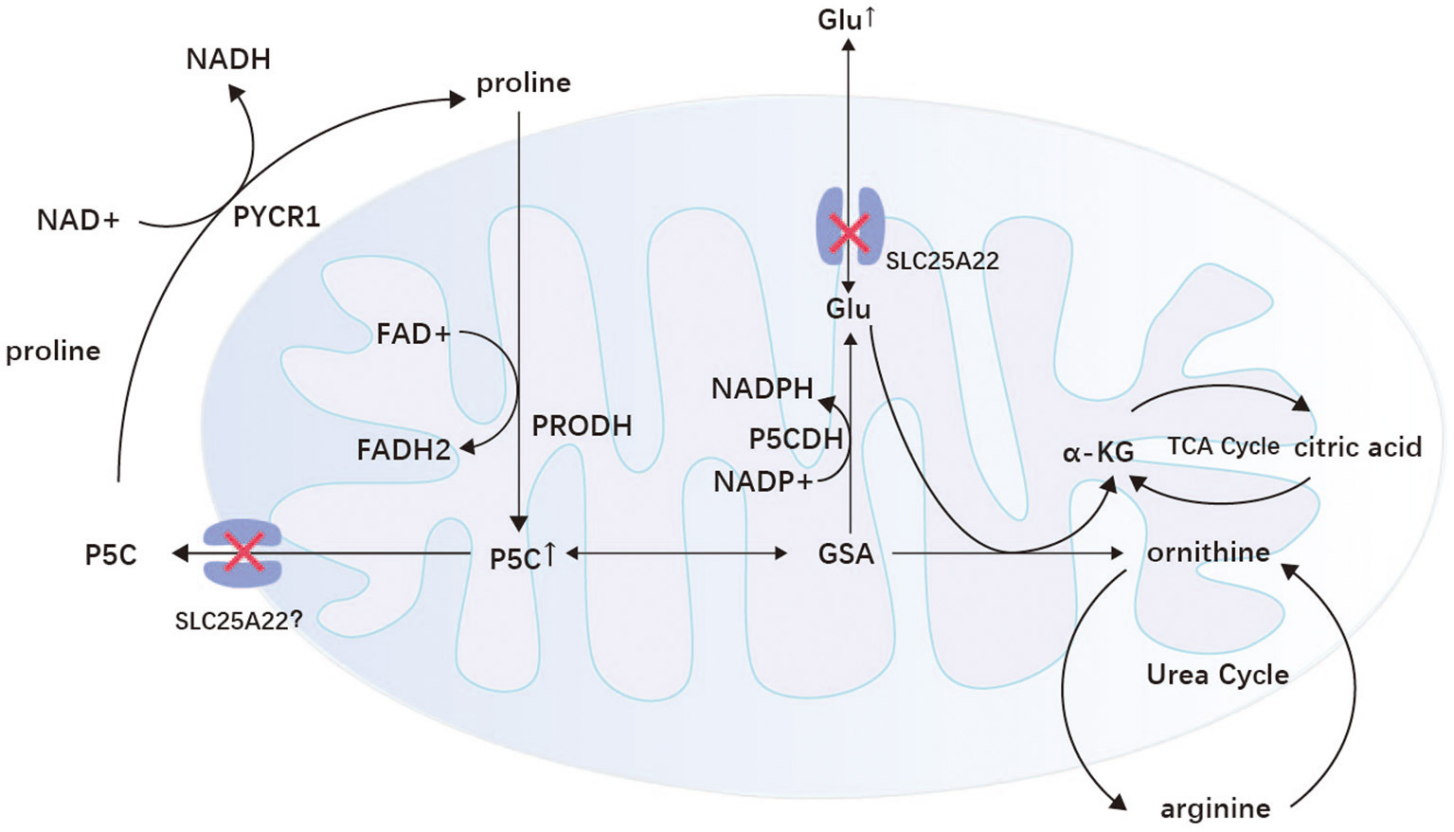
Proline/P5C cycle and P5C metabolism in mitochondria. GC1 is found on the mitochondria of astrocytes and transports glutamate in both directions. When the *SLC25A22* gene is mutated, glutamate transport and the mitochondrial P5C cycle are impaired, leading to abnormal cell excitation and metabolism. P5C, delta (1)-pyrroline-5-carboxylate; GSA, glutamate semialdehyde; α-KG, α-ketoglutaric acid; Glu, glutamic acid; P5CDH, P5C dehydrogenase; PRODH, proline dehydrogenase/proline oxidase; PYCR1, pyrroline-5-carboxylate reductase 1; TCA cycle, tricarboxylic acid cycle.

The SLC25 gene family encodes mitochondrial carriers that transport various metabolites through the inner mitochondrial membrane and hence the name mitochondrial carrier family (102). *SLC25A22* is one of the SLC25 gene isoforms located on chromosome 11p15.5, encoding the mitochondrial glutamate carrier 1(GC1) (103, 104). This protein is expressed only in the brain, mainly on astrocytes, and is an essential channel for glutamate entry into mitochondria (105, 106). With variants of *SLC25A22*, mitochondrial transport of glutamate is impaired. NADPH cannot be transferred to the respiratory chain, resulting in an inadequate supply of intracellular energy and a significant accumulation of glutamate in the cytoplasm, which is released into the synaptic gap, leading to neuronal excitation (105, 107). In addition, the *SLC25A22* variant also leads to an impaired mitochondrial pyrroline-5-carboxylic acid (P5C) cycle, which increases P5C in mitochondria. P5C is eventually further metabolized to glutamate, ornithine, and arginine (108). Therefore, plasma metabolic screening may show elevated levels of these amino acids (107). The excess glutamate produced by the impaired P5C cycle in mitochondria leads to inactivation of the urea cycle, resulting in the production of large amounts of citric acid, and the excess NADPH and citric acid together promote lipid synthesis (Figure 3) (107). Thus, muscle biopsies from patients with *SLC25A22* show a large number of fat vacuoles within the muscle fibers.

#### Clinical presentation

Variants of *SLC25A22* can cause developmental and epileptic encephalopathies type 3 (DEE3) (OMIM 609304), and their clinical manifestations may be consistent with early infantile myoclonic epilepsy, migratory focal epilepsy in infancy, and epileptic spasms (103, 107, 109). The main clinical features are neonatal seizures, developmental delay, low eye pressure, abnormal electroretinogram, microcephaly, hypotonia, and active tendon reflexes. The seizures mostly appear within the first few months of life and are mainly focal and take various forms, including myoclonic, tonic, and tonic–clonic seizures. These patients mostly have a severe epilepsy phenotype and a poor prognosis, but a case with a milder phenotype was reported in which the seizures appeared only at the age of 7 years (107). Molinari et al. (103) reported a case of early myoclonic epilepsy in an infant with autosomal recessive inheritance, characterized by an EEG with a burst suppression pattern and a general slowing of background waves (110, 111). However, in infants with migratory focal epilepsy, the characteristic EEG is epilepsy of multifocal origin (109). Brain MRI examinations of patients with DEE3 always show developmental or structural brain abnormalities, such as varying degrees of brain atrophy, delayed myelin formation, and thinning of the corpus callosum (106, 108).

#### Treatment options

The disease has a poor prognosis with high mortality. Epilepsy caused by the *SLC25A22* pathogenic variant is in most cases unresponsive to antiseizure medications and adrenocorticotropic hormones (ACTHs). A 12-year-old girl with seizures starting on the 15th day of life was treated with valproic acid, vigabatrin, levetiracetam, phenobarbital, ethosuximide, clobazam, and ACTH with poor results (112). Giacominia et al. reported a case of a *SLC25A22* heterozygous mutant whose seizures did not respond to levetiracetam and pyridoxine, but the addition of phenobarbital and topiramate resulted in good control of the seizures (110). In conclusion, there are few specific approaches to treat epilepsy caused by the *SLC25A22* pathogenic variant, and new targeted therapies for this gene are urgently needed.

### *SLC35A2* and developmental and epileptic encephalopathy type 22

#### Pathogenesis

*SLC35A2*, located on chromosome Xp11.23, encodes UDP-galactose transporter (UGT), a nucleotide–sugar transporter protein (113, 114). Under physiological conditions, complex polysaccharides are attached to proteins by N-glycosylation to ensure protein functional and structural stability. UGT transports UDP-galactose into the Golgi lumen as a substrate for protein galactosylation. Variants of *SLC35A2* lead to a decrease in galactose within the Golgi and a deficiency of galactose and sialic acid in the N-glycan branch, disrupting the glycosylation process and resulting in congenital disorders of glycosylation (SLC35A2-CDG) (115, 116).

#### Clinical presentation

Kodera et al. first demonstrated that variants of *SLC35A2* are one of the genetic causes of DEE, which is now classified as developmental and epileptic encephalopathy type 22 (DEE22, OMIM 300896) (117). N-Glycosylation defects caused by *SLC35A2* variants may alter the neurotransmission and excitability of neural circuits, leading to seizures (118). Both X-chromosome dominant inheritance and somatic mosaic mutations can lead to SLC35A2-CDG, which is an age-dependent seizure disorder. All patients present with seizures within the first 6 months of life, mainly infantile spasms (115, 117–120). Notably, over time, the seizure form of epilepsy can change. SLC35A2-CDG cases mostly have severe intellectual disability, hypotonia, and delayed language development, with some patients unable to say meaningful words even by age 12 years (117). These patients mostly have dysmorphic features such as coarse facial features, sunken nipples, shortened limbs, skeletal defects, hip subluxation, and scoliosis (119, 121, 122). Some patients also have tetralogy of Fallot, atrial septal defect, ventricular septal defect, and a small amount of pericardial effusion (119). More than half of the patients have ocular abnormalities such as nystagmus (which may stop with seizure control), severe hypotony, iris heterochromia sign, and retinitis pigmentosa (121–123). In addition, patients may also develop systemic manifestations such as coagulation disorders, immune dysfunction, cardiac dysfunction, and liver failure (124). The two cases of somatic mosaic variants reported thus far were both male patients and both showed mental retardation, developmental delay, shortened limbs, microcephaly, small cerebellum, and cerebral hypoplasia/atrophy compared with X-dominant patients (120). Patients with somatic mosaic variants can be seizure-free and have acute nephrotic syndrome, coagulation disorders, gastroesophageal reflux, esophagitis, and duodenal perforation (120).

A majority of patients with SLC35A2-CDG have an EEG with hypsarrhythmia, with a small percentage having a focal multispine wave, a diffuse spike or sharp and slow-wave complex, and a burst suppression (117, 119, 122). Common MRI manifestations in patients with SLC35A2-CDG include corpus callosum thinning, brain atrophy, delayed myelin formation, and a multifocal inhomogeneous abnormal patchy white matter high signal (121, 124). Abnormal glycosylation of serum transferrin is detectably altered in the presence of glycosylation dysfunction. However, the altered transferrin in patients may return to normal after 2–3 years of age (119, 120).

#### Treatment options

Patients with SLC35A2-CDG with standard treatment of epileptic spasms have temporary seizure control but are prone to recurrence, and most recurrences are unresponsive to antiepileptic drugs and ketogenic diet therapy (117, 119, 122). Only one patient with seizure recurrence had complete seizure control with the same dose of ACTH applied again (117, 122). A patient reported by Yates et al. had seizure control with a ketogenic diet combined with vigabatrin and nitrazepam (119). UGT dysfunction can lead to reduced galactose levels in the Golgi lumen and disrupt the glycosylation process, so galactose supplementation can increase the intracytoplasmic UDP-galactose concentration. A total of 11 patients with SLC35A2-CDG were treated with galactose, which was well tolerated and led to a continuous improvement in clinical symptoms and a significant reduction in seizure frequency (121, 123). Only one patient did not achieve any clinical improvement with galactose supplementation (123).

### *SLC38A3* and developmental and epileptic encephalopathy 102

#### Pathogenesis

The SLC38 family is mainly expressed in actively growing cells throughout the body and is responsible for mediating the transport of sodium-coupled neutral amino acids (125). *SLC38A3* is located on chromosome 3p21.31 and encodes a sodium-coupled neutral amino acid transporter protein (SNAT3), which is mainly responsible for the transport of asparagine, glutamine, and histidine (126). *SLC38A3* is most strongly expressed in the liver, skeletal muscle, and pancreas, followed by the kidney, brain, and heart (127). Among them, SLC38A3 is predominantly expressed in astrocytes and endothelial cells in the brain (128). In the brain, glutamate and GABA in the synaptic gap enter astrocytes *via* EAAT2 and GABA transporter 3 (GAT3), respectively, where they are converted into glutamine. After glutamine is released into the extracellular fluid *via* SNAT3 and SNAT5, it enters neurons *via* SNAT1 and SNAT2 to be metabolized into glutamate and GABA, which are used to replenish depleted neurotransmitters (Figure 1) (126). This metabolic process is known as the GABA–glutamate–glutamine cycle. SNAT3, which is expressed in blood–brain barrier endothelial cells, is also responsible for glutamine transport out of the brain (129). Therefore, variants of *SLC38A3* can lead to increased glutamine levels in the brain, which may reduce the levels of the inhibitory neurotransmitter GABA and the excitatory neurotransmitter glutamate. However, the effect of *SLC38A3* variants on neurons is not clear, and the mechanism leading to DEE needs to be further investigated.

#### Clinical presentation

The disease caused by *SLC38A3* pathogenic variants is currently classified as developmental and epileptic encephalopathy type 102 (OMIM 619881) and was first reported by Marafi et al. (130). Currently, 10 patients were reported with DEE102 from six families, five of which had consanguineous marriages (130). All 10 patients had axial hypotonia, absent speech, and global developmental delay/mental retardation (130). Most patients also had epilepsy, peripheral hypertonia, constipation, and visual impairment (129). Visual impairment includes cortical blindness and cone-rod dystrophy (129). Epilepsy typically presents within the first 15 months of life and manifests primarily as generalized tonic–clonic, followed by tonic, gelastic, focal status epilepticus, and generalized myoclonic–tonic seizures (129). Patients in this group may also present with dysphagia, movement disorder, hyperreflexia, hepatomegaly, gastroesophageal reflex disease, and atrial septal defect. A total of six patients had background slowing of the EEG and epileptiform abnormalities, but no characteristic EEG manifestations have been found (129). MRI is mainly characterized by callosal abnormalities, dysmyelination, and brain atrophy (129). Rarely, thin brainstem, generous extra-axial CSF spaces, dolichocephaly, mild posterior plagiocephaly, and mild superior vermian hypoplasia are present (129). In one patient, cerebrospinal fluid N-acetyl glutamine and N-acetyl aspartate levels were elevated. His brother, who was also affected, had significantly lower plasma glutamine and N-acetylglutamine levels (129). This may be related to the fact that SNAT3 is expressed in the endothelial cells of the blood–brain barrier and is responsible for the transfer of glutamine.

#### Treatment options

*SLC38A3* variant-associated epilepsy is typically unresponsive to multiple antiepileptic drugs. The ketogenic diet has been reported to be only partially effective in patients with DEE102 (130). Vigabatrin is the most effective treatment for reducing the frequency of seizures 3-fold (130). In addition, patients with DEE102 respond positively to benzodiazepines (130). In two patients, not only did the seizures improve with benzodiazepines but also their neurological function remained at baseline levels (130). This is probably because benzodiazepines act on GABAA receptors and promote GABAergic inhibition (131). Vigabatrin irreversibly inhibits GABA transaminase and increases GABA concentrations in the CNS (132). Thus, benzodiazepines and aminocaproic acid partially restored the reduction in GABA due to SNAT3 dysfunction.

## Discussion

In this article, we first proposed the concept of transporter protein disease. We described the nine DEE phenotypes in detail in terms of pathogenesis, clinical features, ancillary tests, and precise treatment (Table 1). All nine DEEs have early-onset refractory epilepsy and global developmental delay, but they can be distinguished based on their characteristic presentation. Targeting the pathogenesis of each specific disease may lead to the development of new antiepileptic drugs to facilitate the treatment of refractory epilepsy. For example, enhanced EAAT2 expression increases glutamate uptake by glial cells and protects neurons from excitatory neurotransmitter damage (12, 18). Recombinant forms of interleukin 1 receptor antagonists, LDN/OSU-0212320, ceftriaxone, and Parawixin10 all act by enhancing EAAT2 expression (18–20). Among them, ceftriaxone has been applied to one clinical patient, but no significant efficacy was found (16, 24, 25). Therefore, more clinical studies are needed to validate the efficacy of ceftriaxone in *SLC1A2*-associated epilepsy.

**Table 1:** Summary of SLC genotypes associated with DEE and their clinical features.

**SLC genotype**	**Encoded protein**	**DEE typology**	**Clinical manifestations**	**Possible treatments**	**References**
SLC1A2	The sodium-dependent glutamate transporter 2	DEE 41	Seizures appear within the first 2 years of life, microcephaly, congenital low eye pressure, cortical visual impairment, generalized developmental delay, and movement disorders.	Anakinra; LDN/OSU-0212320; Parawixin10; Ceftriaxone; tamoxifen; growth factor α-selective agonists	(9, 11, 15, 17, 19–21, 23, 123, 124)
SLC2A1	glucose transporter 1	Not yet included in OMIM network	Seizures, neurodevelopmental delays, microcephaly, dystonia, spasticity, varying degrees of language impairment and complex motor dysfunction such as ataxia	Ketogenic diet; Triheptanoin; adeno-associated virus type 9-mediated gene therapy; Protein transduction domain delivery of therapeutic macromolecules	(28, 45–48, 133, 134)
SLC6A1	Na^+^-Cl^−^coupled γ-aminobutyric acid transporter 1	Not yet included in OMIM network	Seizures, autism spectrum disorders, intellectual disabilities, and language disorders	Ketogenic diet; Valproic acid; Vigabatrin; Tiagabine; 4-Phenylbutyrate; adenovirus-associated vector-mediated gene therapy; Antisense oligonucleotide therapy;	(39, 49–52, 55–57, 122)
SLC12A5	K^+^-Cl^−^ cotransporter 2	DEE 34	Seizures with developmental delay and regression within the first 6 months of life, microcephaly and low eye pressure	potassium bromide; IGF-1; Selective serotonin reuptake inhibitors; WNK-SPAK/OSR1 pathway inhibitor	(62, 65, 70, 71, 75, 77)
SLC13A5	Na^+^-coupled citrate transporter protein	DEE 25	Neonatal epilepsy, developmental delay, dental dysplasia, ataxia, axial hypophthalmia, and dyskinesia	Phenobarbital; valproic acid; Acetazolamide	(84, 86, 87, 90–92)
SLC25A12	mitochondrial aspartate/glutamate transporter	DEE 39	Epilepsy, psychomotor retardation, hypotonia and microcephaly	Ketogenic diet	(48, 96–100, 103, 107)
SLC25A22	Mitochondrial glutamate H^+^ or OH^−^ transporter	DEE 3	Neonatal seizures, developmental delay, hypotonia, electroretinogram abnormalities, microcephaly, peculiar facial features, and myofibrillar fat vacuoles	No specific treatment is available; phenobarbital and topiramate may be effective	(103, 107, 109)
SLC35A2	Golgi uridine diphosphate galactose transporter protein	DEE 22	Seizures, developmental delay, hypotonia, growth defects and deformities, coagulation disorders, immune dysfunction, cardiac dysfunction, liver and kidney failure. Eye manifestations include iris heterochromia, retinal white spots, blindness, and retinitis pigmentosa.	ACTH; galactose supplementation	(117, 121–123)
SLC38A3	Sodium-coupled neutral amino acid transporter	DEE102	Axial hypotonia, absent speech, global developmental delay/mental retardation, Seizures, peripheral hypertonia, constipation, visual impairment, cortical blindness, cone-rod dystrophy, dysphagia, hyperreflexia, hepatomegaly, gastroesophageal reflex disease, and atrial septal defect	Vigabatrin, benzodiazepines	(130)

GLUT1DS1 is typical of DEE, and the degree of clinical symptoms and EEG abnormalities fluctuates with fasting (32). Low cerebrospinal fluid glucose is present in almost all patients with GLUT1DS. The use of alternative energy sources, such as a ketogenic diet, medium-chain triglyceride oil, and triheptanoin, can be effective in improving energy deficiency in the brain (35, 45–47). Further research is still needed to determine the optimal age to start a ketogenic diet. In addition, AAV9-mediated gene replacement therapy is a current research hot spot that has shown positive results in animal models, with the expectation of translating it into clinical treatment for GLUT1DS (50).

SLC6A1-associated epilepsy better responds to drugs that mainly act on increasing GABA concentrations in the brain, such as a ketogenic diet, valproate, tiagabine, and vigabatrin (57, 58, 135). A recent study revealed that 4-phenylbutyrate promotes wild-type GAT-1 transport and increases GABA reuptake by astrocytes and neurons, suggesting the opportunity to promote wild protein transport for therapeutic purposes, and this concept is being tested in a pilot clinical trial (61). Antisense oligonucleotide therapy and AAV9-mediated gene therapy are also potential treatments (59, 60).

DEE34 is predominantly a malignant migratory focal epilepsy in infants (71, 72). The current drugs for DEE34 mainly target NKCC1 and KCC2. Animal experiments have shown that inhibition of both the WNK-SPAK/OSR1 pathway and stimulation of IGF-2 reduce the intracellular chloride concentration and promote the inhibitory function of GABA (78–80). The efficacy of bumetanide as an antiepileptic agent needs to be further validated; it is effective in temporal lobe epilepsy and neonatal epilepsy but does not improve seizures due to neonatal ischemic-hypoxic encephalopathy (73–75).

Patients with *SLC13A5* commonly present with seizures in the first week of life and have characteristic dental hypoplasia and speech changes (86, 87). MRI has characteristic punctate white matter damage (89). There is near normalization of the EEG background after the neonatal period in patients with *SLC13A5* mutations (88). However, further studies are needed to compare the differences between the EEG of patients with well-controlled epilepsy and the normal EEG background of patients with persistent neurocognitive dysfunction (88). Acetazolamide is only effective in some patients, and more studies are expected to discover the specific antiepileptic mechanism of acetazolamide for early application (90, 92).

*SLC25A12* is a severe epileptic encephalopathy with characteristic myelin developmental disorders and a reduced brain N-acetylaspartate content (96, 97). Patients with *SLC25A12* variants mostly have a normal respiratory chain and can use a ketogenic diet to bypass their abnormal metabolism, protect their neurons, and promote myelin formation (99–101).

Due to metabolic impairment, patients with *SLC25A22* can exhibit a large number of fat vacuoles within the muscle fibers and increased plasma ornithine, arginine, and glutamate levels (107). The use of multiple antiepileptic drugs was ineffective in 69% of patients. Only one patient responded well to phenobarbital and topiramate (110). There is a lack of effective treatment for patients with the *SLC25A22* variant, so further research into its pathogenesis is necessary to discover targeted treatment options.

Patients with SLC35A2-CDG have seizure spasms and arrhythmias as their main manifestations, along with severe intellectual disability and some systemic manifestations (119). It is unclear whether congenital heart disease is part of this syndrome. Almost all patients with SLC35A2-CDG return to normal transferrin glycosylation after 3 years of age; therefore, genetic testing should be performed for early diagnosis in patients with a phenotype consistent with SLC35A2-CDG, even if ferritin glycosylation is normal (119, 120). Their seizures can be temporarily controlled with standard therapy for epileptic spasms but are prone to recurrence (117, 119, 122). However, researchers have proposed supplemental galactose therapy for the target of N-glycosylation deficiency, showing good therapeutic effects in current patients (121, 123). A long-term follow-up is still needed to observe its long-term effects.

Patients with *SLC38A3* variants mainly present with axial hypotonia, absent speech, global developmental delay/mental retardation, epilepsy, and visual impairment (130). Decreased plasma glutamine and N-acetylglutamine levels and increased cerebrospinal fluid N-acetylglutamine levels may serve as biomarkers in patients with DEE102 (130). However, not all patients exhibit this metabolic abnormality, so more plasma and cerebrospinal fluid metabolic profiles of patients with DEE102 need to be examined to determine their metabolic changes and to help clarify the mechanism of the disease onset (130). Drugs do not completely control their seizures. Therefore, the underlying mechanism of how *SLC38A3* variants cause DEE needs to be clarified so that targeted treatments can be developed (130).

In conclusion, mutations in nine SLC genes are known to cause DEE, and numerous SLC gene variants are thought to be associated with epilepsy but have yet to be fully validated. SLC is involved in the transmembrane transport of various substances and is a therapeutic target for many diseases. As a result, a thorough investigation of SLC and its pathogenesis could lead to the identification of new precise therapeutic targets, which are critical for developing new approaches to treating refractory epilepsy.

## Author contributions

YG is the main author of the review and completed the collection and analysis of the relevant literature, and the writing of the first draft of the manuscript. ZW, CL, GL, and YF participated in the analysis and organization of the literature. YD was the conceptualizer and the person in charge of the project and directed the writing of the manuscript. All authors contributed to the article and approved the submitted version.

## Funding

This study was funded by the National Key R&D Program of China, Precision medicine program—Cohort study on nervous system disease [Grant number: 2017YFC0907700 (2017–2021)], and National Natural Science Foundation of China (Grant number: 81871007).

## Conflict of interest

The authors declare that the research was conducted in the absence of any commercial or financial relationships that could be construed as a potential conflict of interest.

## Publisher's note

All claims expressed in this article are solely those of the authors and do not necessarily represent those of their affiliated organizations, or those of the publisher, the editors and the reviewers. Any product that may be evaluated in this article, or claim that may be made by its manufacturer, is not guaranteed or endorsed by the publisher.
